# Learned Use of Picture Cues by Bumblebees (*Bombus impatiens*) in a Delayed Matching Task

**DOI:** 10.3390/bs6040022

**Published:** 2016-10-14

**Authors:** Emma Thompson, Catherine Plowright

**Affiliations:** School of Psychology, University of Ottawa, Ottawa, ON K1N 6N5, Canada; ethom091@uottawa.ca

**Keywords:** bumblebee, *Bombus*, delayed matching, picture-object correspondence

## Abstract

Picture-object correspondence provides an alternate method of investigating delayed matching by providing a cue (picture) which may be spontaneously perceived as similar but different from a corresponding target. Memory for, and corresponding choice of, a target corresponding to a cue could be facilitated by the use of a picture. Bumblebees have been found to both easily differentiate images from corresponding objects but also spontaneously perceive a similarity between the two. Herein, an approach was designed to test the possible use of picture cues to signal reward in a delayed matching task. Target choice preference corresponding to picture cues was tested among three bumblebee (*Bombus impatiens*) colonies using photograph cues (presented prior to target stimuli) corresponding to one of four target stimuli. Photograph cues were the only predictor of corresponding target reward, presented in stable locations. Rewarded and unrewarded tests show a choice preference significantly higher than chance for targets matching the cue. Results suggest that bumblebees can learn to use picture cues in a delayed matching task. Furthermore, experience, conditions of reward inconsistency and location, are discussed as possible contributing factors to learning in a delayed matching task.

## 1. Introduction

In a dynamic and noisy world, it is best to attend to reliable cues and when possible disregard those that may be irrelevant, misleading, or at times redundant [1]. The more distantly removed—in space or time—a secondary cue is from a corresponding target, the less likely that cue will reliably convey information about the target. In other words, it is best to rely relatively more on direct target cues and relatively less on indirect cues. However, flexibly attending and responding to different sources of information can provide coping mechanisms in complex environments [2,3]. Bees depend upon floral resources in constant flux of bloom, depletion, and expiration, which provide multiple signal cues, such as color, shape, taste, and size. Additionally, the environment provides many potential indirect or secondary indicators of nearby targets, including: foliage and vegetation; conspecifics and other animals; and airborne scent. Any one of these cues may signal nectar or pollen, but in redundant combinations not all may elicit equal attention.

Secondary cues may be learned as being generally associated with certain types of stimuli or specifically associated with a specific target. General correspondents provide flexible information about common associations among similar types of stimuli, such as the higher likelihood of flowers being located in areas with foliage than areas without. Specific correspondents provide information about individual stimuli, such as the learning of particular landmarks along a route to a known flower patch [4]. Delayed matching tasks test the capacity to see a cue, separated from a target, and then from multiple stimuli select a target or location corresponding to that cue directly or symbolically. The task has been adapted in studies of animal cognition for a variety of species to determine what is remembered and for how long [5]. Bumblebees can match-to-sample (cue remains visible when choosing among stimuli) in a ‘same-different’ task [6]. They can also learn to respond to contextual priming color cues when choosing between two subsequently presented stimuli as long as the pairs of colors were presented at two different places [7]. Honeybees can learn to solve both symbolic [8] and non-symbolic delayed matching to sample (DMTS) tasks including categorization [9,10]. Honeybees are further able to learn to disregard an additional irrelevant cue in a DMTS task, and transfer this learning to a novel cue but only when the relevant cue is provided in a consistent place or position in a sequence [11]. Delayed matching to sample in bumblebees, however, has remained elusive. Possible obstacles include foraging strategies [12] and the abilities in color discrimination and object detection [13] of bumblebees, which differ from those of honeybees. Nonetheless, hints of success, usually by one or a few individuals, suggest that this capacity or variations may yet be elicited in bumblebees under some circumstances.

One significant challenge for bumblebees in tests of DMTS may be the location variation needed to test ‘true’ matching to sample instead of positional learning, similar to landmark cue use. However, this condition is relatively unnatural; while bees may face challenges of flower growth, depletion, and expiration, flowers do not move. In a confined laboratory setting, the constantly changing location of known stimuli may undermine tests of delayed matching. Context can provide necessary discriminating information for bumblebees when faced with a delayed priming task [7] and tasks requiring matching of one cue to a reward and another to their nest [14,15]. Stable location of target stimuli may facilitate cue to target correspondence in a variation of traditional DMTS whereby a non-spatial cue can correspond to a predictable location [16].

The use of a picture cue in a delayed matching task may further facilitate bumblebee performance. Previous findings have determined that bumblebees can both easily differentiate an object from a corresponding picture but also perceive a relation between them [17]. As such, photographs can play a unique role in cue learning: (i) potentially being more likely to trigger memory for, and therefore approach to, the corresponding target; (ii) and lessen the risk that experience with an unrewarding cue will diminish the strength of rewarded training to the target, as may occur when the cue and target are exactly the same. In other words, the subject can learn that the picture of the object (the cue) is unrewarding but the object itself (target) is rewarding, as opposed to the object (cue) seen first being unrewarding but that same object seen later (target) being rewarding. It has been suggested that honeybee performance on DMTS may improve by a ‘win-stay’ approach, whereby honeybees are more likely to approach more familiar stimuli [12,18]. Honeybees and bumblebees display significant differences in foraging strategy and behavior, and it is possible that among bumblebees, a familiar but unrewarding stimulus (cue) could reduce rather than increase the likelihood of approaching the same type of stimulus again (target).

In the present study, photographic cues corresponding to a selection of target stimuli test cue use by bumblebees in an alternative DMTS task with stable target location whereby a cue signals a rewarding target among four stimuli but all four stimuli remain consistently positioned [16]. The photograph cue-target combination was chosen to also facilitate association between cue and target and potential novel transfer to never before seen photograph and object stimuli during testing. Cue-target matching was defined as approaching the object corresponding to a photographic cue immediately following a cue-target combination change. Bees displaying matching at levels above chance during training were subsequently tested for rewarded, unrewarded, and novel preference for targets matching picture cues.

## 2. Materials and Methods

### 2.1. Subjects

Three colonies of commercially raised bumblebees (*Bombus impatiens*) were donated for this study by Koppert Canada. A total of *n* = 20 bees were tested: 10 bees from colony 1, 4 from colony 2, and 6 from colony 3. The bees did not have any experience outside of the colony before the experiment began. The colonies were fed pollen ad libitum and sugar solution (1:2 sugar to water by volume) was available by foraging during experimental training sessions within a radial arm maze each day. Colored number tags were glued to the thorax of each worker to allow for individual identification and monitoring.

### 2.2. Materials

**Figure 1:**
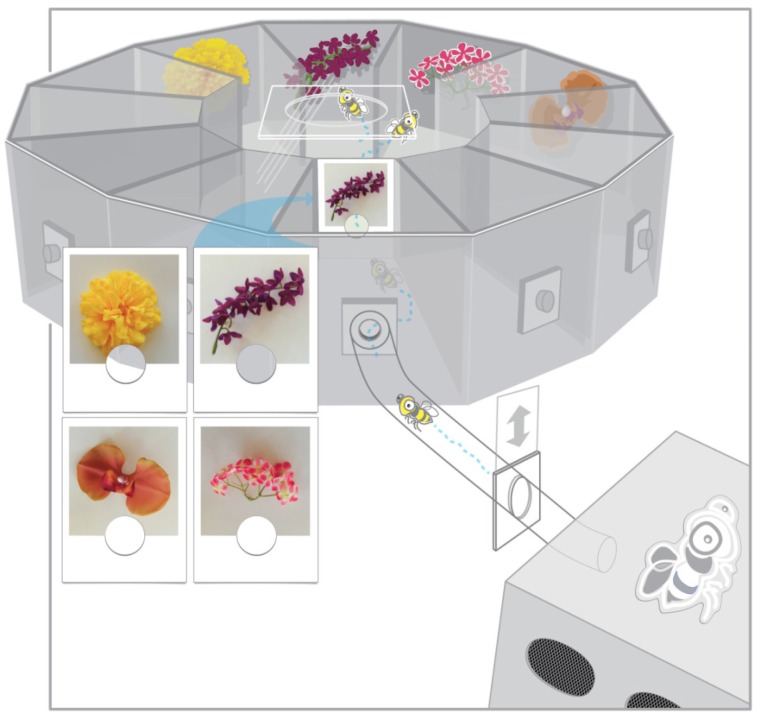
Radial arm maze with connected hive box used in the experiment. A photograph cue was placed on a gated entry way to the center of the maze, requiring bees to fly through or near the image before entering the radial maze. Four of the corridors were open with artificial flowers placed on the end wall of each surrounding a feeder trough. The picture cue viewed upon entering the maze corresponded to a matching object in a stable location within one of the maze corridors rewarded with sugar solution. For the three ‘flowers’ that did not match the photograph cue, the feeder troughs were empty. Figure modified from an original [19] with kind permission from Springer Science and Business Media.

The bees were housed in a plastic nest box attached via a wooden corridor and screen tube to a 12-arm radial maze (corridors were 14 cm long and 15 cm high opening to a central area 17 cm high and 6 cm wide). Bees entered the maze through one corridor, in which a picture-cue was mounted on the gated entry to the central opening of the maze (see Figure 1). The entry gate provided a small hole through which bees entered the maze, randomly flipped so that the entry hole was positioned either in the top or bottom half of the gate. Picture cues were unaltered photographs of the artificial flower stimuli presented in the maze. Photographs were taken with a Panasonic DMC-FZ20 camera and printed on a Canon MP560 ink jet printer [17]. Only the same four corridors and the corridor serving as an entrance were used during the experiment. All remaining corridors were closed with a solid gate. The open corridors contained one of four synthetic fabric ‘flowers’ located on the back wall of the corridor surrounding a feeder trough.

High frequency (>40 kHz) light ballasts (Sylvania Quicktronic T8 QHE4x32T8/112) were used to minimize disrupting behavior due to flicker [20]. Fluorescent bulbs (Sylvania model FO32/841/XP/SS/EC03) hung above the maze.

### 2.3. Procedure

#### 2.3.1. Design

Bees were trained to a delayed matching task wherein a photograph of one of four artificial flower target stimuli was presented prior to entering the center of the maze. The delay in the task could neither be experimentally varied nor controlled: the delay was incurred as the bee travelled from the photograph to one of the corridors harboring an artificial flower. A training trial consisted of a bee entering the maze, foraging from sugar solution reward at one of the four stimuli and returning to the hive. Throughout the experiment, neither data recording nor stimulus presentation were automated. Behavior was monitored by the experimenter in real time. Movement back and forth between the maze and hive was unhindered for bees undergoing training. Additionally, within the maze bees could continue foraging even if they made an incorrect initial choice. A picture cue signaled reward available at the target stimulus corresponding to the picture. The stimuli were positioned at the end of each of the four open corridors, on or near a feeder trough protruding into the maze through the outer wall. During rewarded trials, the feeder trough contained sugar solution for the object corresponding to the picture cue but the remaining three stimuli troughs were empty. The cue-target combinations presented were always chosen randomly. The location of each target stimulus within the maze was kept consistent during training and all testing.

#### 2.3.2. Training

*Colony 1*: It was hypothesized that repeated exposure would be needed for bees to learn the delayed matching task and so training sessions presented cue-target combinations for multiple foraging visits for 20 min before the combination was changed for one of the other cue-target combinations, hereafter referred to as ‘blocked’ trials. Training sessions were conducted for approximately 6 h a day, during which the four cue-target combinations were randomly presented in blocked trials. Bees received training in small groups of approximately five. Training ranged from 3 to 11 days for each bee before individually meeting training criterion for testing. The training criterion used to assess learning of the task required bees to first approach the object corresponding to the picture cue three out of five times immediately following a cue-rewarded target change. When bees repeatedly failed to meet the training criterion, the number of repeated exposures to a cue-target combination, before it was changed, was gradually decreased. The number of repeated exposures within a block of trials was reduced until each cue-target combination was presented for only one foraging visit for every bee. Different cue-target combinations presented for every subsequent trial are hereafter referred to as ‘alternating’ trials. As the time between cue-target changes diminished bees began to pass the training criteria and then proceeded to the testing phase.

*Colonies 2 and 3*: Given that bees from colony 1 only exhibited matching during alternating trials this training method was implemented for both colonies 2 and 3. However, not only did bees not exhibit learning, the activity level of foragers was very low and so the exposure time was stretched into blocked trials. Activity level increased but again bees failed to meet the criterion for delayed matching. Once again, the exposure to each combination was reduced to alternating trials, as with colony 1, and the bees began to meet the training criterion allowing testing for rewarded, unrewarded, and novel matching.

#### 2.3.3. Testing

*Colony 1*: Bees from this colony were only tested under rewarded conditions upon meeting the training criteria. Bees were allowed in the maze only one at a time. They were individually observed for up to 10 test trials (or until activity ceased), during which the picture cue and corresponding reward with the matching object was changed for every visit (as it had been under randomly alternating training trials). The first choice of the bee was recorded, defined as contact made with the object or feeder trough at the end of any open corridor. If the first choice corresponded to the picture cue, this was considered to be a ‘match’ and, if it did not, a ‘non-match’. All picture cue and object combinations were familiar to the bees (the same as those used during training: A, B, D, and E).

*Colonies 2 and 3*: Bees from colonies 2 and 3 were tested for unrewarded matching and novel matching in addition to the original test of rewarded matching. Once the training criterion was met, bees were again observed in the maze, one at a time, for between 4 and 10 subsequent trials, during which reward remained present and the first choice was recorded. The nature of a delayed matching task restricts the number of unrewarded tasks that may be presented; unrewarded trials presented too frequently risk the subject learning that the contingency has become unreliable. Given that a matching task cannot make use of unrewarded testing often, unrewarded tests were conducted approximately every 10 rewarded trials. All picture-cue and object stimuli combinations for rewarded and unrewarded tests were familiar stimuli (A, B, C, and D) used in training. Given the differing activity level of the individual bees, each bee completed 2, 3, or 4 unrewarded tests. One novel unrewarded test was also conducted for 8 of the 10 bees (a drop in activity for the two remaining bees prevented novel testing). The novel test was similar to the familiar unrewarded test but none of the stimuli had previously been exposed to the bees. This novel test could only be presented once per bee before experience may influence subsequent choice, and was also presented following 10 rewarded trials.

### 2.4. Statistical Analysis

A replicated goodness-of-fit test using the *G*-statistic was used to analyze the binary choice proportion data replicated within bees (10 rewarded test choices, 2 to 4 unrewarded test choices, and 1 novel test choice each). *G_P_* value tested whether or not pooled or group proportion differed from a theoretical value of chance (1/4), and *G_H_* for heterogeneity or individual differences. Tests of significance compared the *G* test statistic to a χ^2^ value [21]. A logistic model, using SPSS Statistics for Windows version 22.0 (IBM Corp., Armonk, NY, USA), was fitted to the choice proportions to determine whether cue-target matching depended on which of the four flowers was rewarding.

## 3. Results

Can bees learn to use a picture cue, associated with the corresponding object target, in a delayed matching task?

### 3.1. Colony 1: Rewarded Tests of Delayed Matching

**Figure 2:**
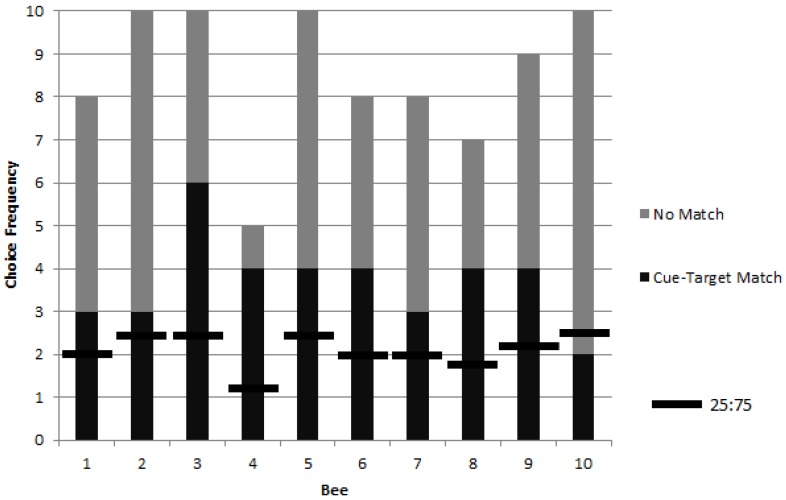
Choice frequencies of rewarded testing for 10 bees in colony 1 showed a significant preference for object stimuli corresponding to a picture cue when compared to a chance value of one in four choices (i.e., a ratio of 25:75). No significant individual differences were found, but the group choice proportion was significantly higher than chance.

Following training, 10 bees exhibited a significant preference to match a photograph cue to a target in a foraging task (Figure 2). Upon meeting the training criterion (see methods) the choice proportions of 10 bees were observed for up to 10 rewarded trials each. The results show a group choice proportion for the object corresponding to the picture cue that was significantly higher than chance (*G_P_* = 13.8, *p* = 0.0002, *df* = 1). Individual differences were not significant (*G_H_* = 8.07, *p* = 0.53, *df* = 9).

**Figure 3:**
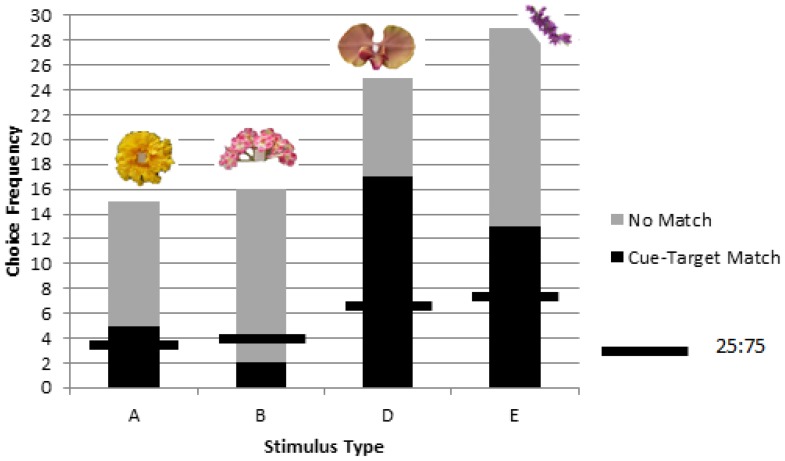
Despite overall significant matching for objects corresponding to picture cues in rewarded testing of colony 1, one stimulus, ‘B’, was found to be matched at significantly lower levels than the other three stimuli (A, D, and E). The chance value of one in four choices is shown as a horizontal line for each stimulus type.

A significant difference was found in matching preference among the stimuli types (A, B, D, and E) (χ^2^ = 11.96, *p* = 0.008, *df* = 3), with the ‘pink inflorescence’ stimulus B being matched at significantly lower levels than the other three stimuli (χ^2^ = 4.72, *p* = 0.03, *df* = 1) (Figure 3).

### 3.2. Colonies 2 and 3: Rewarded, Unrewarded, and Novel Tests of Delayed Matching

**Figure 4:**
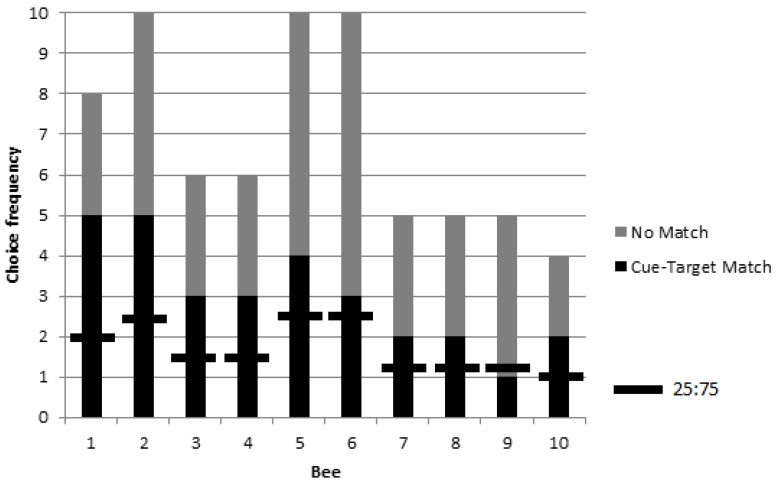
Choice frequencies of rewarded testing for 10 bees in colonies 2 and 3 showed a significant preference for object stimuli corresponding to a picture cue when compared to a chance value one in four choices (i.e., a ratio of 25:75). No significant individual differences were found, but the group choice proportion was significantly higher than chance.

A logistic model revealed no significant effect of colony on choice proportions (χ^2^ = 1.17, *p* = 0.28, *df* = 1) and so the data were pooled (*n* = 10 bees: *n* = 4 from colony 2, and *n* = 6 from colony 3). The group exhibited a higher than chance choice proportion for objects corresponding to a matching picture cue during rewarded testing (*G_P_* = 11.14, *p* = 0.0008, *df* = 1) (Figure 4). Again, no significant individual differences were found (*G_H_* = 3.71, *p* = 0.93, *df* = 9).

**Figure 5:**
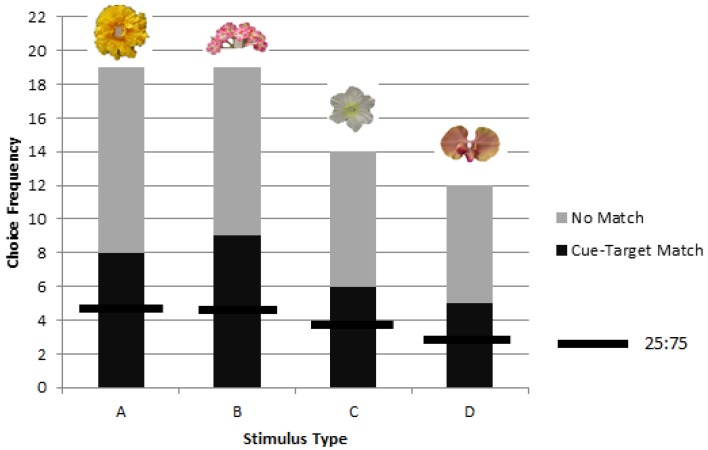
Combined choice frequencies in rewarded testing for colonies 2 and 3 for each stimulus type (A, B, C, and D) showed significant preference for the object corresponding to a picture cue across all stimuli types with no significant differences between A, B, C, or D. The chance value of one in four choices is shown as a horizontal line for each stimulus type.

No significant difference in matching among the four stimuli types (A, B, C, and D) was found during rewarded testing (χ^2^ = 0.20, *p* = 0.98, *df* = 3) (Figure 5).

**Figure 6:**
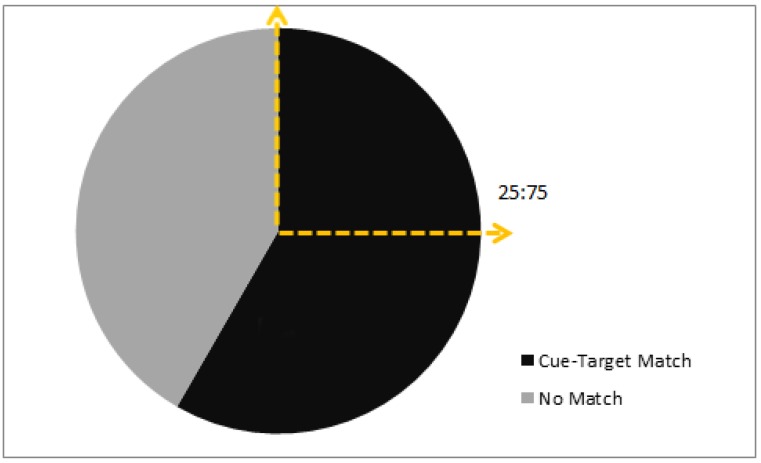
Choice frequencies of unrewarded testing for colonies 2 and 3 showed a significant preference for object stimuli corresponding to the picture cue over non-matching objects; matching occurred 58% of the time with a chance proportion of 25%.

Interspersed between every 10 rewarded trials of the DMTS task an unrewarded test was presented as often as possible for each bee. Unrewarding test trials also showed a choice proportion for object stimuli corresponding to the picture cue significantly higher than chance (*G_P_* = 11.97, *p* = 0.0005, *df* = 1) (Figure 6). No significant individual variation was found (*G_H_* = 13.73, *p* = 0.13, *df* = 9).

Unrewarded novel testing of picture cue matching to objects never before experienced by the bees did not evidence transfer. Eight out of ten bees were each tested once on a novel DMTS task but the choice proportions did not differ from chance, only 2 out of 8 (25%) chose the novel object corresponding to the picture cue.

## 4. Discussion

Bumblebees can learn to use a picture cue to find rewarding stimuli while foraging in a delayed matching task when the stimuli remain in consistent positions. Both rewarded and unrewarded testing showed significant preference for object stimuli matching picture cues. We obtained no evidence that matching would appear to transfer to novel cue-target combinations but the number of choices was limited to a single test trial for each of eight bees, and so there is no claim here to have demonstrated an inability in transfer learning. Without evidence of novel transfer or location change, the present results of delayed matching may correspond to delayed cue-target matching, cue-location matching, or a combination whereby the cue became associated with the location facilitated by picture to target correspondence. In the future, methods used in research on spatial and non-spatial coding of objects by vertebrates might be adapted for use with invertebrates to rule on the question of the contents of learning [22]. In the present study, only the cue predicted which target would be rewarding. Although floral constancy, the tendency to approach relatively more familiar stimuli, could also explain the choice pattern of ‘matching’ corresponding objects to picture stimuli, it is unlikely given that the image cues were never rewarding. Positioning of the cue at the top or the bottom of the gate was random and provided no information about reward. Unrewarded testing of picture cue-target matching was consistent with DMTS proportions found for honeybees [23], and this study could serve as a stepping stone towards a systematic species comparison in bee cognition. However, the bumblebees in the present study may have been using the picture cues as route cues, signaling the location of reward and not necessarily matching the cue and object. In the future, tests with targets placed in novel locations, or new stimuli placed in old locations, might serve to distinguish between these possibilities. The delayed matching task employed in this study retained unchanging position for target stimuli within the maze to reduce the previously suggested difficulty reflected by constantly changing ‘flower’ locations within a small, familiar environment.

Past research had determined that bumblebees perceive both a difference and similarity between pictures and corresponding object targets [17]. The results of the current study further show that pictures can also be used by bumblebees in a delayed matching task. It is possible that the picture cue became associated with both a known location and the corresponding target therein. Multimodal floral signaling can increase learning speed, persistence, and facilitate memory among foragers [24,25]. Secondary or associative cues can further provision serial priming cues to facilitate detection before a target becomes visible [26]. Airborne scent in particular is believed to trigger memory and foraging for the corresponding, but out of sight, flower [27]. While naturally learned secondary cues, specific to a route or location, would not necessarily resemble the target, directed foraging may benefit from using cues similar to the target itself, by triggering memory for the similar, corresponding stimulus. How bees judge the similarity between a 3D flower and its 2D image is not well understood. In our previous research [17], we found that for one flower in particular, used again in the present study (stimulus ‘B’), neither the black and white silhouette nor the drawing of the flower were judged as similar to the object. The image somehow failed to capture some important elements of the object itself. The same may have been true here when colony 1 failed to match stimulus ‘B’ to the cue. Given that another group succeeded, the question of possible colony differences, as documented in other learning tasks [28], also remain to be addressed.

Observations made throughout this study highlight some additional components that may contribute to delayed matching by bumblebees but require further exploration. The present results, in combination with past difficulty training bumblebees to DMTS [12], may suggest additional conditions required for bumblebee learning in a delayed matching task. Below, we speculate how the following aspects of our task design may have contributed to success: (i) a history of foraging experience; (ii) high reward inconsistency; (iii) and stationary location of familiar stimuli.

### *(i)* Experience

Training in the current study required days (from 3 to 11) of exposure before bees exhibited a preference for stimuli matching the photograph cue. A meta-analysis of DMTS among many animals including honeybees [23] suggested that amount of training improves signal to noise differentiation and recall for memories of cues. Other studies have shown that facilitation or pre-training can influence learning of an otherwise impossible task or shift strategic or attentional processes. Experience or facilitated learning has been found to be beneficial or essential in the performance of the bumblebee: reversal learning; use of global over local cues in navigation and rotated image discrimination [12,29,30]. Facilitated learning may well have contributed in this study to experienced bees later exhibiting matching but it cannot be ruled out that any form of foraging experience may facilitate delayed matching. A general history of foraging experience may simply encourage bees to remain active despite high levels of inconsistency and difficulty finding reward.

### *(ii)* Reward Inconsistency

Selective attention best facilitates foraging or hunting for consistent resources but when resources change, either declining or increasing in availability, broadening of attention becomes advantageous [31]. Changing conditions can alter the relative worth of various resources as well as the corresponding sources of information signaling those resources by re-directing attention and reinforcing memories or recognition of alternatives [25,32].

Successful delayed matching by bumblebees in this study may have required conditions of high reward inconsistency. Bumblebees do show flexibility under inconsistent or changing circumstances, and outperform the more persistent honeybee on tasks of reversal learning with repeated experience [12]. In the current study, when the same target was rewarded twice or more in a row (blocked trials), bees exhibited a strong preference for the previously rewarded stimulus, disregarding the cue. However, when the cue and rewarding target combination were changed following every foraging visit for each bee (alternating trials), matching was observed and evidenced by both rewarded and unrewarded testing. Foraging animals often rely on selective attention to best attend to relevant over irrelevant stimuli but still gather information broadly when needed due to resource change, loss, or depletion [31]. It has been found that blue jays used a predictive signal when choosing between two stimuli but, as with the bumblebees in the present study, only when the signal was reliable and under inconsistent environmental conditions [33]. The signal was ignored if unpredictable and the environment was consistent. The possible effect of inconsistent resources influencing delayed matching has also been suggested for the spider *Misumena vatia* [34].

While floral constancy may represent a more energy efficient and focused strategy when compared with sampling, information about alternate resources may still be retained for later use when resource state changes and sampling becomes necessary [35]. In this study, during blocked trials, bees could rely on a relatively successful perseverating strategy because change occurred only following repeated reward with the same stimulus. Only when perseverating was never successful, during alternating trials, did the bees begin to exhibit matching and use of the picture cue.

Lastly, interference may be reduced with increased number of cues and reward variability among cues. Repeated or prolonged exposure to a cue can interfere with acquisition of alternate cues. Greater numbers of stimuli are known to reduce interference in DMTS [16], and picture stimuli changed more frequently may further reduce interference, reducing prolonged exposure to any one stimulus. In other words, the use of four stimuli and corresponding cues for which reward was changed frequently (alternating trials) may have reduced the potential for interference.

### *(iii)* Location

As mentioned, the delayed matching evidenced in this study may correspond to either a cue-target match or a cue-location match. Changing target stimuli location previously resulted in significantly decreased foraging activity, but this does not necessarily suggest that bees were learning a cue-location match. In a confined laboratory space, the movement of stimuli within a familiar environment corresponds poorly with natural challenges of floral cycle. Field study may be better able to replicate stimuli change and thereby test for DMTS without stable location of target stimuli. However, it is also likely that the bees were learning to use the picture cues as spatial cues to direct location or route choice rather than matching the picture to the corresponding object [7,36]. While secondary, environmental cues may have limited value predicting rewarding resources, spatial cues used along a route to rewarding resources are commonly used in bee navigation [7,9,12,36]. Airborne scent cues likewise rely on directing attention to the corresponding flower of a known location to elicit foraging for a specific flower [32].

## 5. Conclusions

The results of the present study do evidence a capacity among bumblebees for learned picture cue use when foraging in a delayed matching task, under conditions of high environmental inconsistency without variation in target location. Experience is likely needed for foraging bumblebees to persist with a task in which only one stimulus is rewarding at a time and the rewarding stimulus changes constantly. This likely corresponds to a preferred reliance on primary cues, those directly presented by the potentially rewarding stimulus (flower color, size, location, etc.), instead of relying on secondary or associated cues, likely to be less predictive or reliable (e.g., presence of foliage). Switching from a preferred to a less preferred strategy has been observed in bee navigation, whereby bees will disregard landmark cues in favor of route memory unless that route memory is unreliable [37]. Similarly, although illumination is most often disregarded as a misleading feature (which changes throughout the day), bees can learn to attend to and use this cue when it is the only predictive cue for reward [38]. The results of the current study likewise suggest that bumblebees prefer alternative strategies to delayed matching but can learn to use cues when foraging if preferred behavior patterns fail in a highly inconsistent environment. Perseveration is a relatively less risky and costly method of foraging when conditions favor repeated visits to the same or similar flowers, but under conditions when perseveration is unsuccessful alternative methods, such as the use of associative cues, could be beneficial.
